# Correction: Prevalence of polypharmacy and the association with non-communicable diseases in Qatari elderly patients attending primary healthcare centers: A cross-sectional study

**DOI:** 10.1371/journal.pone.0238343

**Published:** 2020-08-24

**Authors:** Ayman Al-Dahshan, Noora Al-Kubiasi, Manal Al-Zaidan, Wael Saeed, Vahe Kehyayan, Iheb Bougmiza

The following information is missing from the Funding statement: Open Access funding is provided by the Qatar National Library.
