# Supplementary material for: Prevalence of polypharmacy and the association with non-communicable diseases in Qatari elderly patients attending primary healthcare centers: A cross-sectional study
Source: PLoS One. 2020 Jun 11;15(6):e0234386. doi: 10.1371/journal.pone.0234386 (PMC7289385; doi:10.1371/journal.pone.0234386)
Supplement: Attachment — Submitted filename: RESPONSE TO REVIEWERS_ PONE-D-19-22287 - Final.docx [file pone.0234386.s002.docx]

PONE-D-19-22287
Prevalence of polypharmacy and its associated factors among Qatari elderly patients attending primary healthcare centers, 2017
**RESPONSE TO REVIEWERS**

**General Response:**

We appreciated the many insightful comments made by the reviewers. We studied them carefully and made diligent efforts in revising our manuscript. As a result, we consider our manuscript much improved to the reviewers’ satisfaction.

- **Academic Editor’s Comments:**

**Comment #1:**

In the introduction section, the authors talk about physician-related factors that may contribute to polypharmacy, however, such factors are not included in the study.

**Response:**

Thank you for bringing this issue to our attention. This was not our intent and we can appreciate the reviewer’s concern. Our focus was to study the prevalence of polypharmacy and its association with major non-communicable diseases and other factors (e.g.: gender, age, BMI, etc.) You will note in the revised manuscript that we have removed the reference to “physician-related factors”.

**Comment #2:**

How do you define simultaneous use of five or more medications per person?

**Response:**

Thank you for your comment. By simultaneous use of five or more medications per person” we meant concurrent use of five or more medications. That is, we characterized polypharmacy as “5 or more medications”. This point was addressed in the revised manuscript. **(Methods section: P. 5, line 103-104)**

**Comment #3:**

The authors should also look at polypharmacy as a continuous measure.

**Response:**

Thank you for your suggestion about treating polypharmacy as a continuous variable. We agree that this would be an appropriate option. However, the reason we chose to treat it as a categorical variable was (1) we found in our literature review that most studies were treating it as categorical, and (2) we wanted to be able to compare our findings with others.

**Comment #4:**

Data analysis and results sections are not properly written and are not organized well. There are many grammatical mistakes.

**Response:**

Thank you for this feedback. We have engaged the services of a native English speaker to review the entire manuscript and make the necessary corrections. The data analysis section was modified as advised. **(Method section: P. 6, line 111-118)**

**Comment #5:**

Discussion: “A possible explanation for the high prevalence of polypharmacy found in our study is the fact that elderly patients are being seen and treated by different health care professionals, which might result in adding new prescription, at different occasions.”

If the authors think that continuity of care is an important factor associated with polypharmacy then why was it not included.

**Response:**

Thank you for your observation. At the time of our study, patients attending Qatar’s primary health centers were usually seen by different primary physicians at different visits. However, the family medicine model was recently implemented across all primary health centers in the country and consequently patients are now being seen by their own family medicine physician at each visit. In the discussion section of the manuscript we have added text about the issue of lack of continuity of care. **(Discussion section: P. 12, line 187-191)**

- **Reviewer’s Comments:**

Dear Authors,

“Thank you for give me the opportunity to review this manuscript. It treats an interesting matter for health care workers. I hope my comments will be useful to do that.

The manuscript entitled “Prevalence of polypharmacy and its associated factors among Qatari elderly patients attending primary health centers, 2017” is based on a study that was carried out with the purpose to investigate the prevalence and the associated factors of polypharmacy among the elderly (≥65 years) nationals in this population.

Given increased survival for older individuals, including those with multiple chronic conditions and polypharmacy, the topic has high significance and potential impact. Overall, the manuscript contributes to a better understanding of this issue.

You have performed an interesting work, where the study problem, as well as the different concepts, are clearly described. The backgrounds are exposed and the need to carry out the study is justified. However, some concerns should be addressed. Please see the following questions:”

**Response:** Thank you for this positive comment.

**Comment #1:**

Abstract: P.2 line 33: “Please, provide a definition of the abbreviation BMI”.

**Response:**

A definition of BMI was added (BMI= Body Mass Index). **(Abstract: P. 2, Line 32-33)**

**Comment #2:**

Introduction section: P.5, line 97 Main objective: “There is an inconsistency between the definition that the main objective is made in this section with the one made in the abstract”.

**Response:**

Thank you for bringing this discrepancy to our attention. We have corrected this discrepancy. The objective now reads “to determine the prevalence of polypharmacy (≥ 5 drugs) and its association with non-communicable diseases (NCDs) in elderly (≥65 years) Qatari patients attending Primary Healthcare (PHC) centers in Qatar”. **(Abstract: P.2, line 22-24), (Intro. section: P.4, line 74-76)**

**Comment #3:**

“Given the small number of variables that are taken into account and described in the results, the title of the study can be misleading since the results focus primarily on the relationship between polypharmacy and the main comorbidities observed in the population of study. Therefore, it would be more appropriate for both the title and the objective of the study to refer to the relationship between polypharmacy and such comorbidities”.

**Response:**

The title was modified so it reflects the study objective as follows: (Prevalence of polypharmacy and the association with non-communicable diseases in Qatari ‎elderly patients attending primary healthcare centers: A cross-sectional study‎). **(Title page: P. 1, line 2-3)**

**Comment #4:**

Methods section: P.6 line 116 Study population:

“Reading this section I have raised many doubts. First, it is observed that the study / sample population is made up of the elderly patients (> 65 years old) registered in the Electronic Medical Record (EMR) and who had at least one visit to any primary health center during the study period . This can be a selection bias since the patients who go to the health centers may be the ones with the most pathologies and therefore the most polymedicated. In this case, the results could not be extrapolated to the general Qatari population over 65 years old. What happens to patients not registered in the EMR or who did not go to any health center during the study period? Are they different from those studied?”

**Response:**

The Primary Health Care Corporation is the largest provider of primary care in the State of Qatar. This non-profit organization ‎delivers its services through 23 primary health centers distributed over the country according to population ‎density. Furthermore, PHCs are the common and popular first-line level of contact between community individuals and ‎their healthcare system. The registered number elderly nationals in EMR at the time of the study was **15,286** while the total number of the same population in Qatar in 2017 was **17,895** (according to the planning and statistics authority in Qatar). Thus, this indicates that more than **85%** of Qatari elderly in the country were registered in the EMR.

Overall, findings from this study provide a reliable basis for polypharmacy phenomenon in ‎primary healthcare settings. Therefore, I do agree with the reviewer comment that the study findings could not be generalised to the whole elderly Qatari population, but to Qatari elderly patients attending PHC centers. This point was clarified in the study strengths and limitation section of the manuscript. **(Study strengths and limitations: P. 14, line 221-223; 226-227; 230-231).**

**Comment #5:**

“On the other hand, you talk about medication reconciliation but it is not explained what this procedure consists of as well as the functions of the different members of the multidisciplinary team that participated in it”.

**Response:**

Thank you for this comment. Medication reconciliation is the process of identifying and listing of patients’ most current medications that they are taking in comparison with all the medications that their physicians have prescribed throughout the course of the patients’ treatment. ^(1)^

The multidisciplinary team‎ involved in medication reconciliation includes the primary care physician, the nurse and the pharmacist. Their function is to ensure that the medication list is appropriately reviewed and verified (at different stages of patient encounter) to reduce possible medication errors such as duplicate medication orders or over/under doses. ‎This point was clarified in the manuscript. **(Methods: P. 5, line 95-101).**

*1. Institute for Healthcare Improvement. Medical Reconciliation to Prevent Drug Events. 2020; retrieved from [*[*http://www.ihi.org/Topics/ADEsMedicationReconciliation/Pages/default.aspx*](http://www.ihi.org/Topics/ADEsMedicationReconciliation/Pages/default.aspx)*]*

**Comment #6:**

Definition and Measures: P6 line 122.

“Polypharmacy is defined as the simultaneous use of 5 or more medications per patient. While this definition of polypharmacy is the most frequently described in the literature, as you explain in the introduction, however, this is a poor and very variable definition that would explain the high prevalence of polymedication that you have found in your research. If we want to take into account the possible negative effects that polymedication can have on the health of patients, a definition of polymedication that takes into account the time variable should be used”.

**Response:**

We appreciate this comment. However, we based our definition of polypharmacy on a 2017 systematic review of 111 studies that was conducted to identify and summarize ‎polypharmacy definitions in existing literature ^(2)^. The most commonly reported definition of ‎polypharmacy (80.4%) was the numerical definition of five or more medications daily; moreover, only ‎‎10% of all included studies used numerical definitions of polypharmacy which also included a ‎duration of treatment in the definition.

In our study, it was not feasible to obtain information ‎on the duration of treatment. As a result, we decided to use the numerical definition of five ‎or more medications daily‎. Also, using such a definition, we were able to compare our findings to many others who used the same definition. To further illustrate this, we have now constructed a new table that will show the most frequently (top 20) used medications by patients exposed to polypharmacy. The table demonstrates that most prescriptions were aimed to treat chronic conditions (medications for long-term use). **(Results: P. 8, line 136-141); (Discussion: P. 13, line 195-201)**

*2. Masnoon N, Shakib S, Kalisch-Ellett L, Caughey G. What is polypharmacy? A systematic review of definitions. BMC Geriatr. 2017;17(1).*

**Comment #7:**

“Furthermore, if one of the objectives of the study is to investigate the associated factors of polypharmacy, it would be advisable to include more variables than those studied such as educational level, socio-economic level, marital status, place of residence, number of prescribers, number of visits to health services, etc. In addition, the section on material and methods should include a section where these variables are described and categorized, both dependent and independent variables. Otherwise both the objective and the title of the study should be changed”.

**Response:**

Thank you for this insightful comment. We recognize that this is a limitation of our study. Considering that the data were obtained from the EMR, some variables were not consistently recorded for most patients and thus we were unable to include them in the analysis. **(Study strengths and limitations: P. 14, line 228-230)**

Also, both the objective and the title of the study were modified as advised. **(please see our responses to comments #2 and 3)**

**Comment #8:**

Ethical considerations: P.7 line 149.

“There is no mention that the consent of patients has been obtained to be included in the study by signing an informed consent”.

**Response:**

Thank you for this comment. This study was a retrospective study using data from patients’ electronic medical record. All data were anonymized. The Primary Health Care Corporation-Independent Ethics Committee (PHCC-IEC) waived the requirement to obtain any informed consent since the data used in this study were anonymized (‎reference number PHCC/RS/17/11/015). ‎ This point was clarified in the manuscript. **(Methods: P. 6, line 120-124).**

**Comment #9:**

Results section:

Table 1. Please provide a definition of the different categories of the Body Mass Index variable”.

**Response:**

A definition for each BMI category was added as follows: Underweight (<18.5)‎, Normal (18.5-24.9)‎, Overweight (25-29.9)‎, Obese (≥30)‎. **(Results: Table 1, P. 7)**

**Comment #10:**

Study strengths and limitations section

“The fact that the way in which the sample of the study has been selected may imply a selection bias should be included in this section, as well as the lack of convenience of generalizing the results obtained to the general Qatari population older than 65 years”.

**Response:**

We agree with your comment. **(Please see our response to comment #4)**

**Comment #11:**

Conclusions section

“The conclusions should be more concrete, referring exclusively to the proposed objectives and be supported by the results obtained. Recommendations and personal reflections that are not supported by concrete results should be included in another section of the manuscript such as discussion, prospective or recommendations.”

**Response:**

Thank you for this insightful observation. The conclusion section was revised as suggested. **(Abstract: P. 2, line 35-39); (Conclusions section: P. 14-15, line 233-242)**
