# Supplementary material for: Prevalence of polypharmacy and the association with non-communicable diseases in Qatari elderly patients attending primary healthcare centers: A cross-sectional study
Source: PLoS One. 2020 Jun 11;15(6):e0234386. doi: 10.1371/journal.pone.0234386 (PMC7289385; doi:10.1371/journal.pone.0234386)
Supplement: Attachment — Submitted filename: Responses to Reviewers.docx [file pone.0234386.s003.docx]

**Response to reviewers**

**General Response to the Reviewers’ Comments:** We are appreciative of the insightful comments raised by the Academic Editors. We believe we have responded to each of their comments. Where necessary, we have made revisions in the manuscript. These revisions are tracked. The line numbers referred to in our responses below refer to those in the tracked version of the manuscript.

**ACADEMIC EDITORS:**

We have concluded that a major revision should be performed for the paper to be published according to Plos One criteria. Moreover a bit more speculation might be useful to suggest strategies for clinical improvement or further investigations.

**Comment #1:** The main point is represented by external validity. It seems clear that the data are not representative of general elderly population in Qatar: prevalence of obesity >50%, diabetes almost 80%, asthma 20% are largely greater than population estimates around the world, and may clearly explain the high prevalence of polypharmacy – almost double in comparison with international estimates **(to be cited)**: 39% in US according to ref 18, 44% in Sweden according to Morin L et al., Clin Epidemiol, 2018).

**Response:** Thank you for your insightful observation, I agree with you that the prevalence of polypharmacy in our study is higher than some international figures. This could be explained by the high prevalence of NCDs in our study population. We cited Morin on lines 67 and 206. In the discussion section please see the statement about the association between polypharmacy and prevalence of NCDs (lines 243-244). Our findings are comparable to other regional figures. For example, the prevalence of polypharmacy was 55% in Saudi Arabia and 60% in Kuwait. Also, a population-based study in Korea found that 86% of elderly patients were exposed to polypharmacy.

**Comment #2:** In fact Authors state: "The study population included all Qatari elderly patients (≥65 years) who attended PHC centers during a period of six months from April 1 to September 30, 2017, and who had medication reconciliation done".

What proportion of older Qataris attend PHC?

**Response:** Thank you for your comment. At the time of the study, the total number of elderly nationals who were attending PHC centers (proven by having active electronic medical records/ encounters) was **15,286.** While the total number of the same population in Qatar in 2017 was **17,895** (according to the planning and statistics authority in Qatar). Therefore, this indicates that the proportion of older Qataris who attended PHC centers at the time of the study is almost **85%**. Please see additional response in our response to Comment #4.

**Comment #3:** Which percentage of older patients who attended the PHC underwent a medication reconciliation?

**Response:** According to the Primary Health Care Corporation (PHCC) policy, all patients attending PHC centers must have medication reconciliation. ‎This policy was introduced in January 2017 as a quality measure because patients were seen by any available ‎physicians at the time of their visit‎. Therefore, all patients in our sample have undergone medication reconciliation.

**Comment #4:** Is the sample at least representative of older subjects who attend a Primary Health Center?

**Response:** Thank you for this comment. We believe that our sample is representative of older patients who attend PHC centers because our sample consists of about 37% (5,629/15,286) of the total older Qataris who attend PHC centers. In addition, the gender distribution of all elderly Qataris who attended PHC centers across the country in ‎‎2017 was as follows: males= 7526/15286 (49.2%), females= 7760/15286 (50.8%). This is not ‎greatly different from the gender distribution in our sample (i.e., males= 46.2%; females= 53.8%).‎ Therefore, findings from this study provide a reliable basis for the polypharmacy phenomenon in ‎‎primary healthcare settings‎.

**Comment #5:** If this is the case, **conclusions** should be moderated, stating that the prevalence of polypharmacy of older subjects ATTENDING A PHC in Qatar is very high, and **discussion** may be enriched adding information and perspectives on PHCs.

**Response:** Thank you for this insightful feedback. We modified the discussion and conclusion sections as advised. Please see line 197 for revisions in the discussion and line 270-271 in the conclusion. Regarding information and perspectives of PHCs, please see lines 93 – 100, and 111 – 113.

**Comment #6:** Was there any evaluation of adherence?

**Response:** Thank you for this insightful comment. This is a valid point as our study relied on medications prescribed and reconciled, but not on adherence. To measure adherence would require a dedicated study. We have identified this as a limitation of our study. Please see lines 261 - 265.

**Comment #7:** Was there potential to examine potential drug-drug interactions, ADR or inappropriate use?

**Response:** Thank you for this comment. We did not examine these in our study. However, we (the authors) are currently working on another research to assess the inappropriate use of medications among the same elderly population.

**Comment #8:** Could the difference of polypharmacy between the sexes be explained by differential attendance at PHC? Or to different socio-economic condition?

**Response:** Thank you for this insightful observation. During the period of our study, the attendance of females was higher than their male counterpart (females= 53.8%; males= 46.2%). We controlled for this factor in our logistic regression. However, the difference of polypharmacy between the sexes could be explained by the statistically higher prevalence of all NCDs among females than males except for CVD. In addition, the proportion of those who had “three or more NCDs” was higher among females in ‎comparison to males (60.1% vs. 52.3%; p-value <0.001). Also, obesity was more prevalent among females.‎ Please see the new Table 2 below and in the manuscript.

| **Variable** | **Male** | **Female** | ***p-value*** |
| --- | --- | --- | --- |
|  | **Count (%)** | **Count (%)** |  |
| **No. of chronic conditions**  Zero  One  Two  Three or more | 131 (5.0)  390 (15.0)  719 (27.6)  1361 (52.3) | 113 (3.7)  354 (11.7)  742 (24.5)  1821 (60.1) | <0.001* |
| **Body Mass Index (kg/m^2^)**  Underweight (<18.5)  Normal (18.5-24.9)  Overweight (25-29.9)  Obese (≥30) | 57 (2.2)  411 (15.8)  867 (33.3)  784 (30.1) | 33 (1.1)  194 (6.4)  574 (18.9)  1657 (54.6) | <0.001* |
| **Hypertension**  Yes  No | 2085 (80.1)  518 (19.9) | 2530 (83.4)  505 (16.6) | 0.001* |
| **Diabetes mellitus**  Yes  No | 1893 (72.7)  711 (27.3) | 2283 (75.2)  752 (24.8) | 0.017* |
| **Dyslipidemia**  Yes  No | 966 (37.1)  1638 (62.9) | 1479 (48.7) 1556 (51.3) | <0.001* |
| **Cardiovascular disease**  Yes  No | 664 (25.5)  1940 (74.5) | 505 (16.6)  2530 (83.4) | <0.001* |
| **Asthma**  Yes  No | 430 (16.5)  2174 (83.5) | 722 (23.8)  2312 (76.2) | <0.001* |
| **Arthritis / Osteoarthritis**  Yes  No | 394 (15.1)  2210 (84.9) | 639 (21.1)  2396 (78.9) | <0.001* |
| **Gastroesophageal reflux disease**  Yes  No | 287 (11.0)  2317 (89.0) | 421 (13.9)  2614 (86.1) | 0.001* |
| **Mental disorders**  Yes  No | 57 (2.2)  2547 (97.8) | 120 (4.0)  2915 (96.0) | <0.001* |

**Comment #9:** Have you got information regarding frequency of attendance to PHC as a possible marker of poorer control of comorbid conditions/greater polypharmacy?

**Response:** Thank you for this question. This would have been a valuable information. A research study in Canada showed a significant association between polypharmacy and higher frequency of family physician visits in elderly patients. Please see lines 221-222.

**Comment #10:** Is there an urban rural discrepancy?

**Response:** Thank you for this question. According to PHCC’s Department of Operations, the vast majority of Qatari population 98.75% (15094/15286) who attended PHC centers live in urban areas. This applies to our study population, where around 98.5% lives in urban areas. Thus, we can conclude that there is no urban/rural discrepancy.

**Comment #11:** Is any information available regarding geriatric syndromes (malnutrition, impaired mobility, falls), nursing home placement, and hospitalizations? It would be of interest to assess the association with polypharmacy, controlled for comorbid conditions.

**Response:** Thank you for this comment. As you will note in our manuscript, we controlled for comorbid conditions in the multivariate logistic regression analysis. Regarding to the other variables you mention, as our data source was the electronic medical records PHC centers, such information is not captured.

**Comment #12:** Please add a brief description of PHC: are patients attached to an individual physician or is this a “drop in” type system with multiple prescribers?

**Response:** Thank you for this comment. Please see lines 93 - 96 for a general description of PHCs; and lines 96 – 99 for a specific description of patient/physician assignments.

**Comment #13:** Is the PHC on an electronic medical record viewable by all and are there prescribing reminders according to condition?

**Response:** Yes. EMR is viewable by all healthcare professionals who primary care physicians, nurses, and pharmacists. Please see lines 99 – 100. However, there are no prescription renewal reminders according to the health condition.

**Comment #14:** The authors might usefully refer to Hughes et al., Age Aging, 2013 which examines the potential for polypharmacy in the presence of more than one chronic condition when physicians are adhering to prescribing guidelines.

**Response:**

Thank you for suggesting the work of Hughes et al. We have referred to his work and another source in lines 265-266.

**Journal Requirements:**

**1.)** When submitting your revision, we need you to address these additional requirements: Please ensure that your manuscript meets PLOS ONE's style requirements, including those for file naming. The PLOS ONE style templates can be found at <http://www.plosone.org/attachments/PLOSOneformatting_sample_main_body.pdf> and

<http://www.plosone.org/attachments/PLOSOne_formatting_sample_title_authors_affiliations.pdf>

**Response:** We have checked the style used in our manuscript against those required by PLOS ONE.

**2.)** We note that you have included the phrase “**data not shown**” in your manuscript. Unfortunately, this does not meet our data sharing requirements. PLOS does not permit references to inaccessible data. We require that authors provide all relevant data within the paper, Supporting Information files, or in an acceptable, public repository. Please add a citation to support this phrase or upload the data that corresponds with these findings to a stable repository (such as Figshare or Dryad) and provide and URLs, DOIs, or accession numbers that may be used to access these data. Or, if the data are not a core part of the research being presented in your study, we ask that you remove the phrase that refers to these data.

**Response:** The phrase **“Data not shown”** is misleading. We meant to say that we are not showing the descriptors in lines 169 – 172 in a table. We have removed this phrase. Please see line 172.

**3.)** In the ethics statement in the manuscript and in the online submission form, please provide additional information about the patient records used in your retrospective study. Specifically, please ensure that you have discussed whether all data were fully anonymized before you accessed them and/or whether the IRB or ethics committee waived the requirement for informed consent. If patients provided informed written consent to have data from their medical records used in research, please include this information.

**Response:** Please see lines 139 – 141 where we have added this required information.
